# A complementary dual-platform analytical strategy for resolving matrix effects and stability limitations in bioanalysis

**DOI:** 10.3389/fvets.2026.1951616

**Published:** 2026-09-07

**Authors:** Jang Mi Han, Jungho Yoon, Young Beom Kwak

**Affiliations:** 1 Institute of Digital Anti-Aging Healthcare, Inje University, Brain Korea 21 (BK21) FOUR Project, Gimhae, Republic of Korea; 2 Equine Clinic, Jeju Stud Farm, Korea Racing Authority, Jeju, Republic of Korea; 3 College of Pharmacy, Inje University, Gimhae, Republic of Korea

**Keywords:** equine plasma, famotidine, HPLC, omeprazole, omeprazole sulfone, pharmacokinetics, UHPLC-MS/MS

## Abstract

**Introduction:**

Famotidine and omeprazole are widely used for the treatment of acid-related disorders in racehorses, and their combined administration is expected to provide complementary therapeutic effects due to their distinct mechanisms of action. However, their markedly different physicochemical properties present substantial challenges for simultaneous quantification in biological matrices. This study aimed to develop and validate complementary analytical strategies for the simultaneous determination of famotidine and omeprazole in equine plasma using high-performance liquid chromatography (HPLC) and ultra-high-performance liquid chromatography-tandem mass spectrometry (UHPLC-MS/MS).

**Methods:**

Dual-platform analytical methods were developed, with sample preparation conditions tailored to the physicochemical properties of each compound. For HPLC analysis, the effects of sample preparation conditions on the chromatographic behavior and linearity of famotidine and omeprazole were evaluated. For UHPLC-MS/MS analysis, a pH-neutral sample preparation strategy was established to preserve the stability of acid-labile omeprazole. The developed methods were validated according to relevant analytical validation parameters and subsequently applied to equine plasma samples collected following drug administration for pharmacokinetic evaluation.

**Results:**

In HPLC analysis, famotidine exhibited pH-dependent peak splitting associated with changes in its ionization state, which was resolved by acidifying the sample preparation solvent. Omeprazole showed limited solubility in aqueous environments, resulting in nonlinearity at higher concentrations; this was overcome by acidifying the plasma matrix, enabling quantification over 100-5,000 μg/mL. Although the HPLC method effectively characterized the analytical behavior of both compounds, its sensitivity was insufficient for trace-level pharmacokinetic analysis. Therefore, a UHPLC-MS/MS method was developed using pH-neutral sample preparation, enabling the simultaneous determination of omeprazole and its metabolite, omeprazole sulfone. The UHPLC-MS/MS method achieved an LLOQ of 50 ng/mL, representing an approximately 2,000-fold improvement in analytical sensitivity compared with HPLC. The developed methods demonstrated acceptable validation performance and were successfully applied to equine plasma samples, enabling the determination of concentration-time profiles and key pharmacokinetic parameters.

**Discussion:**

The findings demonstrate that differences in the physicochemical properties and stability of famotidine and omeprazole require platform-specific sample preparation strategies for reliable simultaneous quantification. The complementary use of HPLC and UHPLC-MS/MS provided both characterization of compound-specific analytical behavior and sensitive quantification of trace drug concentrations in equine plasma. This analytical framework may support pharmacokinetic studies of famotidine and omeprazole in equine models.

## 1 Introduction

Histamine-2 receptor antagonists (H2RAs) and proton pump inhibitors (PPIs) are the primary pharmacological agents used for the treatment of acid-related disorders, such as gastroesophageal reflux disease (GERD) and peptic ulcers (1). Famotidine, a representative H2RAs, competitively binds to histamine H2 receptors located on gastric parietal cells, thereby blocking the interaction between histamine and its receptors (2). Through this mechanism, it inhibits gastric acid secretion mediated by the cyclic adenosine monophosphate (cAMP) signaling pathway (3). Omeprazole, a PPI, functions by irreversibly inhibiting H+/K + -ATPase (the proton pump) in the gastric parietal cells to suppress acid secretion (4, 5). As a prodrug, omeprazole is protonated in the acidic environment of the gastric parietal cell canaliculi, where it is converted to its active sulfenamide form (6). The activated omeprazole forms a covalent bond with cysteine residues of the H+/K + -ATPase within the secretory canaliculi of parietal cells, resulting in the irreversible inactivation of the enzyme and subsequent inhibition of acid secretion (4, 7). Because omeprazole inhibits the final common pathway of acid secretion stimulated by all secretagogues-including histamine, acetylcholine, and gastrin-it provides potent and sustained suppression of gastric acid secretion (8, 9). Furthermore, due to its irreversible binding to the proton pump, omeprazole offers the advantage of sustained efficacy for at least 24 h with a single daily dose (10). However, its onset of action is relatively slow, with maximal acid suppression achieved only after 2 to 3 days of administration, and it is associated with nocturnal acid breakthrough (4, 11, 12). Additionally, omeprazole is associated with drug–drug interactions related to cytochrome P450 (CYP) 2C19 metabolism (13–15). In contrast, while famotidine exhibits a weaker acid-suppressive effect compared to PPIs, it possesses the advantage of a rapid onset of action, suppressing gastric acid within minutes of administration (16). Moreover, famotidine can compensate for the limitations of omeprazole by maintaining effective acid suppression during the nocturnal period (17). Pharmacokinetically, famotidine shows minimal inhibition of CYP enzymes, indicating a negligible risk of related drug interactions (18). Therefore, given that famotidine and omeprazole inhibit gastric acid secretion via distinct mechanisms, their co-administration is anticipated to yield synergistic therapeutic effects. A previous study involving healthy volunteers investigated the potential of co-administering famotidine and omeprazole (19). The combined administration of these two drugs raised the intragastric pH above 4 within one hour. Furthermore, the time required for intragastric pH to reach 4 or higher was significantly shorter, and the proportion of time during which gastric pH was elevated was significantly greater compared with omeprazole monotherapy. These findings suggest that the combination of the sustained efficacy of omeprazole and the rapid onset of famotidine yields superior clinical outcomes compared to the administration of either drug alone.

Gastric ulceration, the primary therapeutic target of famotidine and omeprazole, represents a significant health concern in racehorses. Previous studies have indicated that the prevalence of gastric ulcers in the racehorse population exceeds 70%, reaching as high as 100% in certain cohorts (20–22). Affected horses may suffer from severe health deterioration, manifesting as abdominal pain, recurrent colic, anorexia, and consequent weight loss (23, 24). Therefore, establishing effective therapeutic strategies is imperative for both the treatment of gastric ulcers and the prevention of their recurrence. To this end, the development and validation of a robust analytical method for the simultaneous quantification of famotidine and omeprazole in equine plasma are essential to facilitate pharmacokinetic (PK) studies aimed at evaluating the efficacy of their co-administration.

However, numerous high-performance liquid chromatography (HPLC) and ultra-high-performance liquid chromatography tandem-mass spectrometry (UHPLC–MS/MS) methods have been reported for the quantification of famotidine and omeprazole, either individually or in combination with other pharmaceutical agents (25–40). Conversely, studies focusing on the simultaneous determination of both famotidine and omeprazole remain limited, largely due to the distinct physicochemical properties of the two compounds, and have been primarily restricted to human matrices (19, 41). In equine medicine, where the therapeutic potential of this combination has yet to be fully explored, the lack of a validated method for simultaneous quantification in equine plasma has hindered the investigating of such co-administration strategies.

Therefore, in this study, HPLC and UHPLC–MS/MS methods were developed and validated for the simultaneous determination of famotidine and omeprazole in equine plasma. Famotidine exhibits ionization characteristics that may affect its chromatographic behavior and ionization response, particularly depending on pH, whereas omeprazole is unstable under acidic conditions. Therefore, applying the same sample preparation and analytical conditions to both analytes may be challenging, thereby presenting analytical difficulties. These differences may make it difficult to establish a single set of analytical conditions capable of providing reliable quantification of both analytes across the relevant concentration range. These compounds exhibit distinct physicochemical and analytical properties, including differences in ionization characteristics and chemical stability, making it challenging to establish a single set of analytical conditions for reliable quantification across a broad concentration range. To address these challenges, HPLC and UHPLC–MS/MS were independently optimized according to their respective analytical characteristics and implemented as a complementary dual-platform strategy HPLC was used for the analysis of relatively high concentrations, whereas UHPLC–MS/MS was used for the analysis of relatively high concentrations, whereas UHPLC–MS/MS was used for trace-level quantification and pharmacokinetic applications. Rather than treating these compound specific properties solely as analytical constraints, we addressed them through a complementary dual-platform strategy in which the respective strengths of HPLC and UHPLC–MS/MS were integrated into a unified workflow. This complementary approach enabled the respective strengths of each platform to be utilized for analyzing different concentration ranges within a unified analytical strategy.

## 2 Materials and methods

### 2.1 Chemicals and reagents

Famotidine, omeprazole, and omeprazole sulfone were purchased from Sigma-Aldrich (St. Louis, MO, USA). Phenacetin, used as the internal standard (ISTD) for UHPLC–MS/MS analysis, was also obtained from the same supplier. HPLC-grade methanol (MeOH) and acetonitrile (ACN) were acquired from Burdick & Jackson (Muskegon, MI, USA). Distilled water (DW) was purified using a RO & UP water Purification System (Human Corp., Republic of Korea). Formic acid (FA) and dimethyl sulfoxide (DMSO) were purchased from Sigma-Aldrich (St. Louis, MO, USA), and LC–MS grade FA was used for UHPLC–MS/MS analysis.

### 2.2 HPLC analysis

#### 2.2.1 Preparation of the stock solution

Individual stock solutions of famotidine and omeprazole for HPLC analysis were prepared at a concentration of 200 mg/mL by dissolving each reference standard in DMSO. Subsequently, a mixed stock solution was prepared by combining the prepared individual stock solutions in a 1:1 (v/v) ratio, resulting in a mixed stock solution containing 100 mg/mL of each analyte.

#### 2.2.2 Preparation of the working solutions and calibration standards

To enhance analyte solubility and improve chromatographic peak stability, blank plasma was acidified to a pH of approximately 2 by mixing with FA at a ratio of 9:1 (v/v) prior to use. Working solutions for the construction of calibration curves and quality control (QC) were prepared by diluting the previously prepared mixed stock solution with DMSO. The concentrations of the working solutions for the calibration standards were set at 2, 4, 10, 15, 30, 40, and 100 mg/mL, while the working solution for the QC sample was prepared at 20 mg/mL. Finally, calibration standards and QC samples were prepared by spiking the respective working solutions into the pH-adjusted blank plasma (pH 2.0) at a dilution factor of 20. The resulting final concentrations in the plasma ranged from 100 to 5,000 μg/mL (100, 200, 500, 750, 1,500, 2000, and 5,000 μg/mL) for the calibration standards, and the concentration for the QC sample was established at 1000 μg/mL.

#### 2.2.3 Sample extraction

Sample preparation for HPLC analysis was carried out using protein precipitation (PPT). ACN containing 5% FA was added to the plasma samples at a ratio of 1:3 (v/v). The mixture was vortex-mixed vigorously and subsequently centrifuged at 13,500 rpm for 3 min. The resulting supernatant was collected and immediately subjected to HPLC analysis.

#### 2.2.4 Instruments and analytical conditions

HPLC analysis was performed using a Hitachi Chromaster system (Hitachi, Tokyo, Japan). Chromatographic separation was achieved on a reversed-phase CAPCELL PAK C18 UG120 (5 μm, 4.6 mm I. D × 250 mm, OSAKA SODA, Osaka, Japan) maintained at 30 °C. The flow rate was set at 1.0 mL/min, and the injection volume was 10 μL. The mobile phase consisted of DW (solvent A) and methanol (solvent B). The gradient elution program was established as follows: 0–2 min, 20% B; 2–5 min, 20–50% B; 5–16 min, 50–80% B; 16–16.1 min, 80–20% B; and 16.1–20 min, 20% B for re-equilibration. Detection was performed at wavelengths of 280 nm for famotidine and 310 nm for omeprazole.

#### 2.2.5 Data analysis

Chromatographic data acquisition and processing were performed using the ChromAssist Data Station software (Hitachi, Tokyo, Japan). This software was utilized for peak integration, construction of calibration curves, regression analysis, and quantitative evaluation. Statistical calculations for method validation parameters were conducted using Microsoft Excel 2019 (Microsoft, Redmond, WA, USA).

### 2.3 UHPLC-MS/MS analysis

#### 2.3.1 Preparation of the stock solution

Individual stock solutions of famotidine, omeprazole, and omeprazole sulfone for UHPLC–MS/MS analysis were prepared at a concentration of 300 μg/mL by dissolving each reference standard in DMSO. Subsequently, a mixed stock solution was prepared by combining these individual stock solutions in a 1:1:1 (v/v/v) ratio, resulting in a final concentration of 100 μg/mL for each analyte.

#### 2.3.2 Preparation of the working solution

Working solutions for the construction of calibration curves were prepared by diluting the mixed stock solution with DMSO to obtain concentrations of 5, 10, 20, 30, 50, 80, and 100 μg/mL. Separate working solutions for QC samples were prepared at concentrations of 15, 40, and 75 μg/mL. Finally, calibration standards and QC samples were prepared by spiking blank plasma with the respective working solutions at a dilution ratio of 1:100. The resulting calibration range in plasma was 50, 100, 200, 300, 500, 800, and 1,000 ng/mL, while the QC samples were established at final concentrations of 150, 400, and 750 ng/mL.

#### 2.3.3 Sample extraction

Sample preparation for UHPLC–MS/MS analysis was carried out using PPT. ACN fortified with phenacetin as the ISTD at a concentration of 100 ng/mL was employed as the precipitation solvent. This solvent was added to the plasma samples at a ratio of 1:3 (v/v). The mixture was vortex mixed vigorously and subsequently centrifuged at 13,500 rpm for 3 min to precipitate proteins. The resulting supernatant was collected and immediately subjected to UHPLC–MS/MS analysis.

#### 2.3.4 Instruments and analytical conditions

UHPLC–MS/MS analysis was performed using a Shimadzu Nexera XS ultra-high-performance liquid chromatography (UHPLC) system coupled to an LCMS–8060NX triple quadrupole mass spectrometer (Shimadzu, Kyoto, Japan). The UHPLC system was equipped with a degassing unit (DGU-405), a solvent delivery module (LC-40D xs), an autosampler (SIL-40C xs), a column oven (CTO-40C), a system controller (SCL-40). Chromatographic separation was achieved on a reversed-phase Luna® Omega C18 100 Å (1.6 μm, 100 × 2.1 mm; Phenomenex, Torrance, CA, USA) maintained at 40 °C. The mobile phases consisted of 0.1% FA in DW (solvent A) and 0.1% FA in MeOH (solvent B). The flow rate was set at 0.3 mL/min, and the injection volume was 5 μL. The gradient elution program was established as follows: 0–1 min, 10% B; 1–5 min, 10–90% B; 5–5.1 min, 90–10% B; and 5.1–7 min, 10% B for re-equilibration. The mass spectrometer was operated in electrospray ionization (ESI) positive mode. The key parameters for the ion source were set as follows: nebulizing gas flow, 3 L/min; heating gas flow, 12 L/min; and drying gas flow, 10 L/min. The interface, desolvation line (DL), and heat block temperatures were maintained at 350 °C, 280 °C, and 400 °C, respectively. Quantification was performed in multiple reaction monitoring (MRM) mode. The specific MRM transitions (precursor ion → product ion) and collision energies (CE) for each analyte were as follows: famotidine, *m/z* 338.5 → 189.1 (CE − 30 eV); omeprazole *m/z* 346.2 → 136.2 (CE − 30 eV); omeprazole sulfone *m/z* 362.4 → 77.2 (CE − 50 eV); phenacetin (ISTD) *m/z* 180.1 → 110.0 (CE − 20 eV).

#### 2.3.5 Data analysis

Data acquisition and processing for UHPLC–MS/MS were performed using LabSolutions and LabSolutions Insight LCMS software (Shimadzu, Kyoto, Japan). This software suite was utilized for peak integration, the construction of calibration curves, and quantitative evaluation through regression analysis. Calculations for method validation parameters were performed using Microsoft Excel 2019 (Microsoft, Redmond, WA, USA). Furthermore, PK parameters were calculated using non-compartmental analysis (NCA) via PKSolver (Version 2.0, 2010), an add-in for Microsoft Excel 2019.

### 2.4 Validation for HPLC method

#### 2.4.1 Selectivity

Selectivity was evaluated by analyzing five replicates (*n* = 5). The assessment involved comparing the chromatograms of blank equine plasma with those of samples spiked at the lower limit of quantitation (LLOQ) to determine the presence of potential interfering substances at the retention times of the analytes.

#### 2.4.2 Linearity

Linearity was assessed using seven concentration levels: 100, 200, 500, 750, 1,500. 2000, and 5,000 μg/mL. Each concentration level was analyzed in quadruplicate (*n* = 4). Calibration curves were constructed based on the peak areas of the analytes, and linearity across the entire concentration range was evaluated using the coefficient of determination (R^2^).

#### 2.4.3 Accuracy and precision

Accuracy and precision were evaluated at the QC concentration of 1 mg/mL. The assessment was performed on an intra-day basis with four replicates (*n* = 4).

#### 2.4.4 Recovery

Extraction recovery was determined at the QC concentration level with four replicates (*n* = 4). It was calculated by comparing the peak areas of analytes spiked into plasma before extraction (pre-extraction spiked sample) with those spiked into blank plasma extracts (post-extraction spiked samples).

#### 2.4.5 Stability

Stability was evaluated under three distinct conditions using QC samples. Short-term stability was assessed by analyzing samples stored for 7 days (*n* = 4). Freeze and thaw stability was evaluated after three cycles of freezing at −20 °C and thawing at room temperature (*n* = 4). Autosampler stability was determined by analyzing samples kept in the HPLC autosampler for 24 h (*n* = 3).

#### 2.4.6 Carryover

Carryover was assessed by injecting a blank plasma sample immediately following the analysis of the highest calibration standard (5 mg/mL). This procedure was repeated four times (*n* = 4). Carryover was calculated as a percentage by dividing the peak area observed in the blank sample by the peak area of the highest concentration standard.

### 2.5 Validation for UHPLC-MS/MS method

#### 2.5.1 Selectivity

Selectivity was assessed by analyzing five replicates (*n* = 5). Chromatograms of blank equine plasma were compared with those of samples spiked at the LLOQ to evaluate the presence of potential interfering substances at the retention times of the analytes.

#### 2.5.2 Linearity

Linearity was evaluated at seven concentration levels, including the LLOQ (50, 100, 200, 300, 500, 800, and 1,000 ng/mL). Each concentration level was analyzed in triplicate (*n* = 3). Calibration curves were constructed based on the peak area ratios of the analytes, and linearity across the entire concentration range was assessed using R^2^.

#### 2.5.3 Accuracy and precision

Accuracy and precision were evaluated at four concentration levels, including LLOQ (50 ng/mL), lower quality control (LQC, 150 ng/mL), middle quality control (MQC, 400 ng/mL), and high-quality control (HQC, 750 ng/mL). Intra-day accuracy and precision were determined by analyzing four replicates (*n* = 4) on the same day, while inter-day accuracy and precision were assessed over three consecutive days (*n* = 3).

#### 2.5.4 Recovery

Extraction recovery was evaluated at the LLOQ, LQC, MQC, and HQC concentration levels. The analysis was performed in quadruple (*n* = 4) by comparing the peak areas of samples spiked before extraction (pre-extraction spiked samples) with those spiked into blank plasma extracts (post-extraction spiked samples).

#### 2.5.5 Carryover

Carryover was assessed by injecting a blank plasma sample immediately following the analysis of the highest calibration standard (1,000 ng/mL). This procedure was repeated four times (*n* = 4). Carryover was calculated as a percentage by dividing the peak area observed in the blank sample by the peak area of the highest concentration standard.

#### 2.5.6 Matrix effect

The matrix effect was investigated at the LLOQ, LQC, MQC, and HQC concentration levels with four replicates (*n* = 4). It was evaluated by comparing the peak responses of analytes spiked into post spiking-extraction blank plasma with those of analytes spiked into the pure solvent.

### 2.6 Animals and study design

The study was conducted using four horses (males and females) with body weights of 325, 394, 400, and 515 kg, respectively. The animal experiment was designed primarily as a proof-of-concept (PoC) evaluation to assess the applicability of the developed analytical methods to equine plasma samples and to obtain preliminary pharmacokinetic profiles of famotidine and omeprazole. The animals were randomly assigned to either the single-administration group or the co-administration group. In the single-administration group (*n* = 2), famotidine was administered as a single intravenous (IV) injection at a dose of 0.4 mg/kg. In the co-administration group, famotidine was administered intravenously at the same dose, while omeprazole was concurrently administered orally (PO) at a dose of 4 mg/kg. Blood samples were collected into vacutainer tubes containing lithium heparin at pre-dose (0 h) and at 0.5, 1, 1.5, 2, 2.5, 3, 5, and 8 h post-administration. The collected blood samples were centrifuged at 3000 × g for 5 min. The resulting plasma was separated, transferred to plastic tubes, and stored at −20 °C until analysis. All animal experiments were conducted in accordance with protocols approved by the Institutional Animal Care and Use Committee of the Korea Racing Authority (KRA IACUC-2502) and performed in compliance with the institutional guidelines for the care and use of laboratory animals.

## 3 Results

### 3.1 Optimization of the HPLC method

#### 3.1.1 Famotidine peak optimization

**Figure 1:**
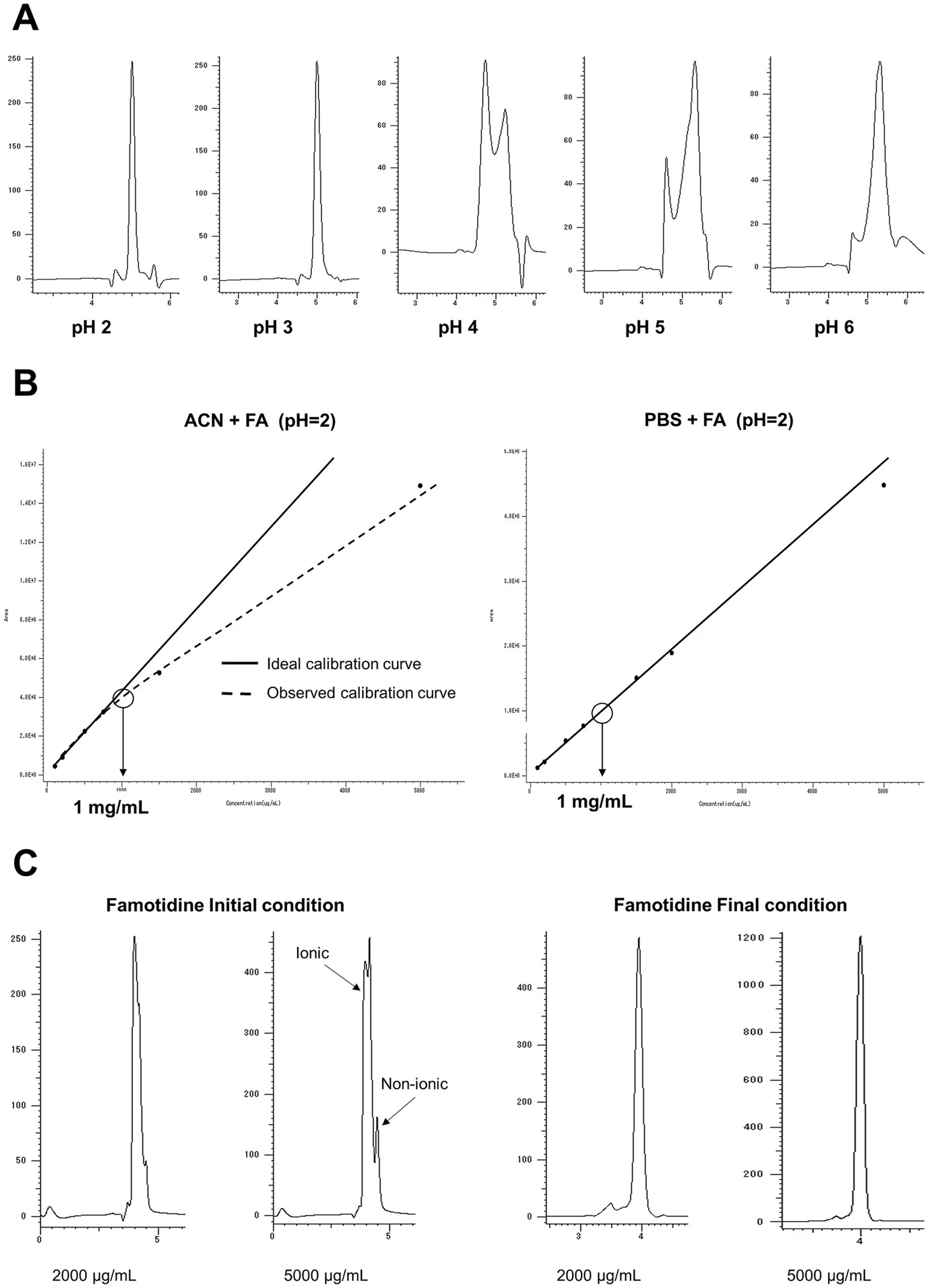
Optimization of sample preparation conditions for the improvement of peak shape and solubility. **(A)** Effect of protein precipitation solvent (ACN) pH (2–6) on the chromatographic peak shape of famotidine. **(B)** Optimization of plasma pH conditions to improve the solubility of omeprazole. **(C)** Resolution of famotidine peak splitting via additional optimization of plasma pH.

During the preliminary HPLC analysis, peak splitting of famotidine was observed. To address this issue, the pH of PPT solvent (ACN containing FA) was adjusted in a stepwise manner from 2 to 6, and the resulting chromatographic changes were evaluated. The results indicated that peak splitting was most pronounced at pH 4. However, adjusting the pH away from this value caused the split peaks to converge into a single peak. As the pH was increased from 4 to 5 and 6, the split peaks shifted to the right and merged. However, they retained a broad shape, rendering them unsuitable for quantitative analysis. Conversely, as the pH was decreased from 4 to 3 and 2, the peaks shifted to the left and coalesced into a sharp, distinct single peak. This transition was accompanied by an increase in peak intensity, indicating improved sensitivity. Accordingly, a pH of 2 was established as the optimal condition for sample preparation in this study, as it provided optimal peak shape and sufficient sensitivity for quantitative analysis (Figure 1A).

#### 3.1.2 Omeprazole solubility and extraction optimization

Although the acidification of the PPT solvent (ACN) successfully resolved the peak splitting of famotidine, a deterioration in the linearity of the omeprazole calibration curve was observed under these conditions. Specifically, at concentrations exceeding 1 mg/mL, the slope of the calibration curve decreased sharply, deviating from the ideal linear model. To mitigate this issue, experiments were conducted to evaluate the impact of the timing of acid addition. Two approaches were compared, the conventional method of adding FA to the PPT solvent and adjusting the pH of the matrix itself by adding FA directly. Phosphate-buffered saline (PBS) was used as a surrogate matrix for plasma to verify the effect of matrix pH adjustment. The results indicated that the conventional method (acidified solvent) still resulted in a non-linear curvature at high concentrations. In contrast, lowering the matrix pH by directly adding FA to PBS, followed by extraction with neutral ACN, restored linearity across the entire concentration range. Consequently, the sample preparation protocol was initially modified to adjust the pH of the plasma sample directly by adding FA, rather than acidifying the PPT solvent (Figure 1B). However, when this modified protocol was applied to achieve linearity for omeprazole, peak splitting of famotidine was observed again, although it had been resolved during the earlier method development phase. To simultaneously ensure the optimal peak shape for famotidine and the linearity of the omeprazole calibration curve, the protocol was finally optimized to adjust the pH of both the plasma sample and the PPT solvent. Application of this final protocol resulted in a sharp, single peak for famotidine without splitting and maintained linearity for omeprazole up to the high concentration range (Figure 1C).

#### 3.1.3 Optimized HPLC method

Based on the optimization results, the final sample preparation method for HPLC analysis was established as follows. Plasma was mixed with FA at a ratio of 9:1 (v/v) to acidify the matrix to a pH of approximately 2. Sample preparation was then initiated by spiking the analytes into this acidified plasma. For the subsequent PPT step, ACN containing 5% FA was employed as the extraction solvent. The precipitation was performed by mixing the acidified plasma with the extraction solvent at a ratio of 1:3 (v/v).

### 3.2 Optimization of the UHPLC–MS/MS method

#### 3.2.1 Adaptation of sample preparation from HPLC to UHPLC-MS/MS

**Figure 2:**
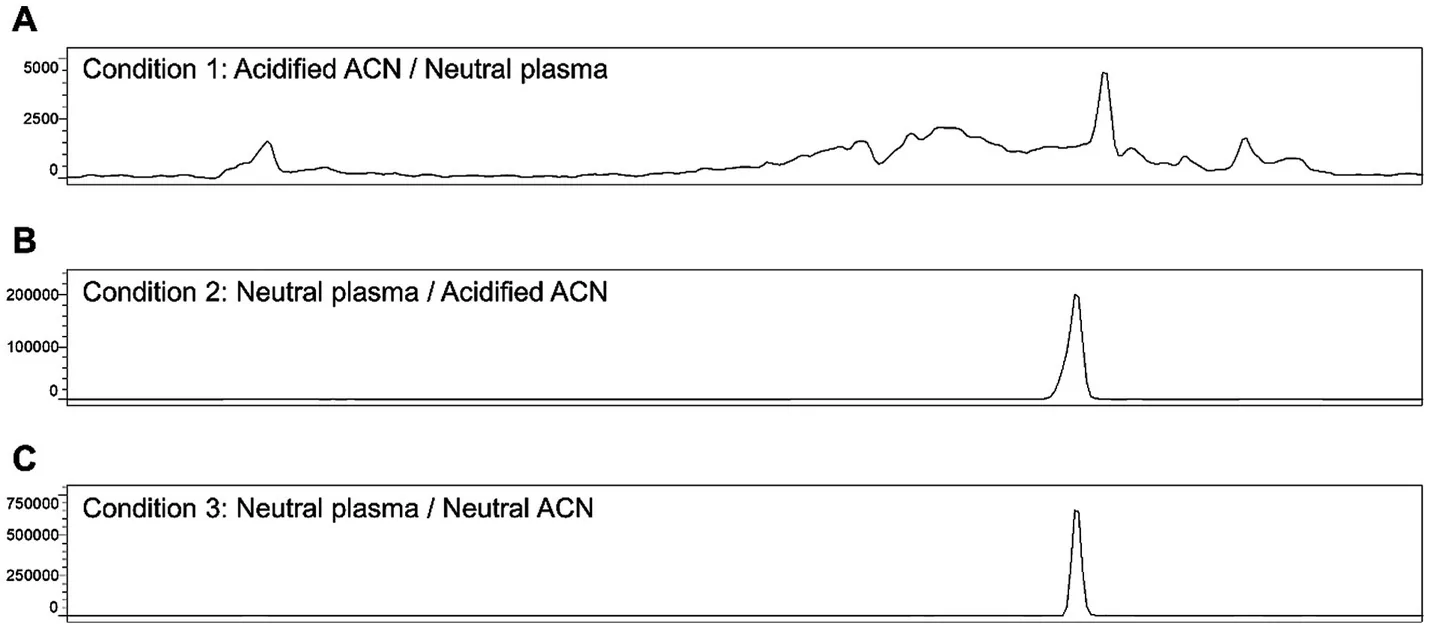
Effect of acid treatment during sample preparation on the stability and chromatographic behavior of omeprazole. Representative chromatograms of omeprazole obtained under three different extraction conditions: **(A)** Acidified plasma with neutral acetonitrile (ACN), **(B)** Neutral plasma with acidified ACN, and **(C)** Neutral plasma with neutral ACN.

Initially, sample preparation for UHPLC–MS/MS was performed under acidic conditions by adding FA to both the plasma and the PPT solvent, following the protocol established for the HPLC method. However, when this protocol was applied to the UHPLC–MS/MS analysis, significant signal instability was observed. Although omeprazole was detectable immediately after sample preparation, a significant loss of signal occurred during continuous analysis sequences or after extended waiting periods. To address this instability and ensure the integrity of omeprazole, a comparative evaluation was conducted under three distinct conditions regarding pH adjustment, Condition 1 (Acidified plasma and neutral ACN), Condition 2 (Neutral plasma and acidified ACN), and Condition 3 (Neutral plasma and neutral ACN). The results demonstrated that omeprazole was undetectable under Condition 1. In Condition 2, although omeprazole was detected, the signal intensity was significantly lower compared to the neutral conditions, and peak fronting was observed in the chromatogram. Conversely, Condition 3 yielded the highest signal intensity and exhibited optimal peak shape. Consequently, in contrast to the HPLC method, a neutral preparation condition without acid addition to either the plasma matrix or the extraction solvent was established as the final sample preparation protocol for UHPLC–MS/MS analysis. This approach provided optimal omeprazole stability and enhanced analytical sensitivity (Figure 2).

#### 3.2.2 Optimized UHPLC-MS/MS method

Based on the optimization results, the final sample preparation protocol for UHPLC–MS/MS analysis was established under neutral conditions. Sample preparation was initiated by spiking the analytes into non-acidified plasma. For the subsequent extraction step, PPT was performed using ACN without acid additives. The ratio of plasma to extraction solvent was maintained at 1:3 (v/v).

### 3.3 Method validation for HPLC

**Figure 3:**
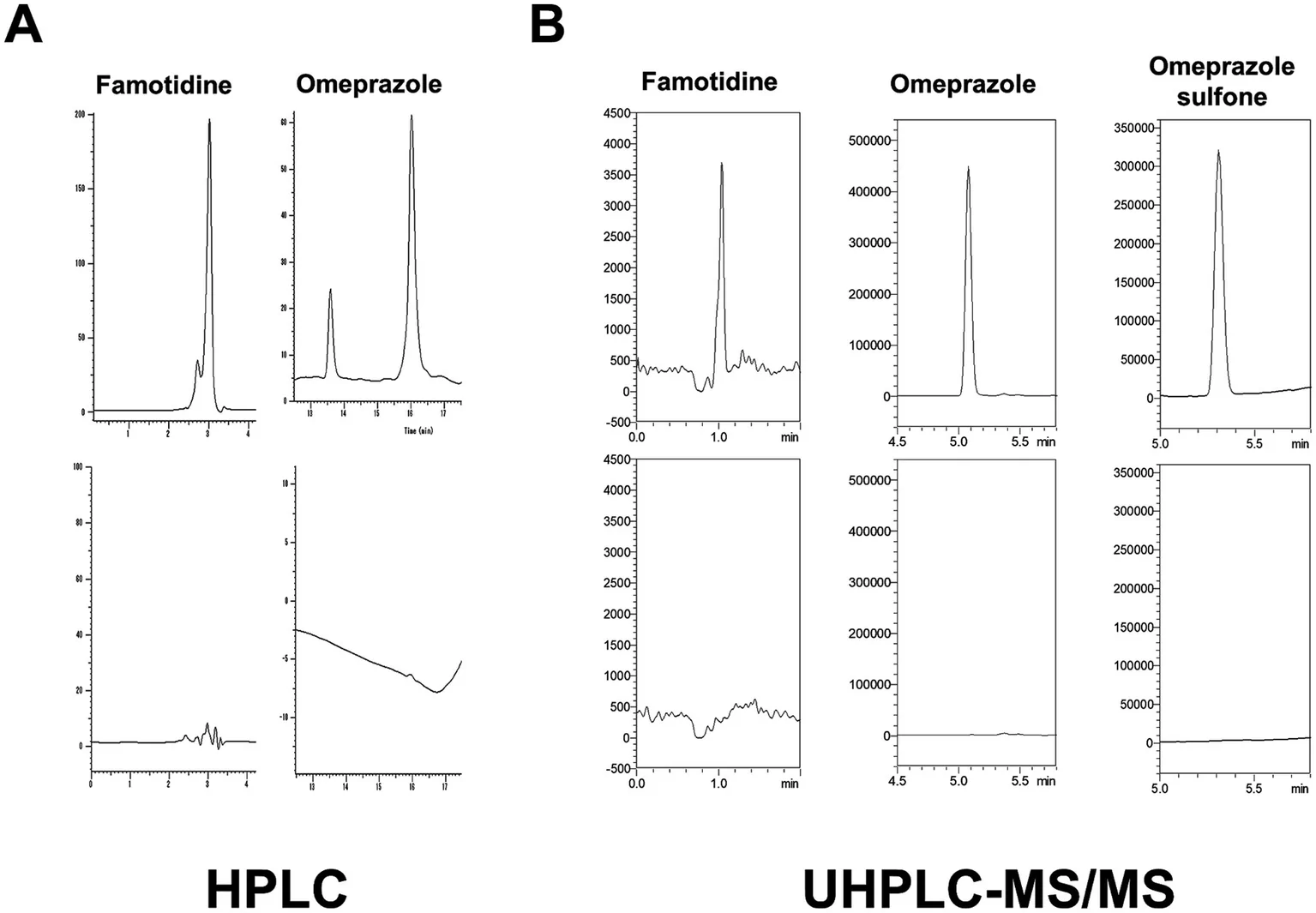
Selectivity for the determination of analytes in equine plasma using **(A)** HPLC and **(B)** UHPLC-MS/MS.

In the selectivity assessment, no significant interfering peaks from endogenous matrix components were observed at the retention times of famotidine and omeprazole. This indicated that the developed method possessed sufficient selectivity for the determination of both analytes in equine plasma (Figure 3A). Regarding linearity, the method exhibited acceptable linear relationships for both drugs, with R2 of 0.993 for omeprazole and 0.997 for famotidine. The accuracy and precision results demonstrated high reliability, with values of 97.4 ± 6.5% for famotidine and 102.3 ± 0.4% for omeprazole at the QC level. Stability evaluations yielded results ranging from 100.4–101.3% for short-term storage, 100.9–110.9% for freeze and thaw cycles, and 109.8–132.9% for autosampler stability. Extraction recovery was found to range from 73.6–119.3%. The observed carryover may increase the analyte signal in subsequent samples and consequently affect the quantitative results, potentially compromising the accuracy and precision of quantitative analysis. No additional analytical conditions were applied to minimize the potential impact of carryover during sample analysis. Potential approaches for reducing carryover, including enhanced autosampler needle washing and additional blank injections, are discussed in the Discussion section (Table 1).

**Table 1:** Validation results for the determination of famotidine and omeprazole in equine plasma using HPLC.

Analyte	LLOQ* (μg/mL)	Nominal concentration (μg/mL)	Intra-day (*n* = 3)		Recovery ± RSD (%)	Carryover ± RSD (%)	Stability (%)		
			Precision (%)	Accuracy (%)			Short-term (7 day)	Freeze and thaw (3 cycle)	Auto sampler (24 h)
Famotidine	100	1,000	6.5	97.4	73.6 ± 7.3	0.8 ± 34.7	100.4	100.9	132.9
Omeprazole	100	1,000	0.4	102.3	119.7 ± 10.1	38.5 ± 13.7	101.3	110.9	109.8

LLOQ*, Lower limit of quantification.

### 3.4 Method validation for UHPLC-MS

In the evaluation of selectivity, no significant interfering peaks from endogenous plasma matrix components were observed at the retention times of famotidine, omeprazole, and omeprazole sulfone. This confirms the high selectivity of the method for the simultaneous determination of these analytes in equine plasma (Figure 3B). Linearity was achieved for all analytes, with mean R2 of 0.995 for famotidine, 0.995 for omeprazole, and 0.995 for omeprazole sulfone. The accuracy and precision results indicated high reliability. For intra-day validation, the accuracy and precision ranges for famotidine were 88.0–101.2% and 1.0–9.3%, respectively. Omeprazole and omeprazole sulfone exhibited accuracies of 92.0–98.4% and 96.2–106.5%, with precisions of 0.7–4.5% and 0.6–4.3%, respectively. For inter-day validation, famotidine exhibited accuracy of 94.0–101.0% with precision of 0.9–5.6%, omeprazole showed 100.4–106.3%, accuracy and 0.9–11.9% precision, and omeprazole sulfone demonstrated 99.6–107.0% accuracy with 1.4–8.3% precision. Extraction recovery was determined to be 32.7–38.4% for famotidine, 25.2–34.7% for omeprazole, and 24.4–31.6% for omeprazole sulfone. No carryover was detected for any of analytes. Matrix effect were calculated to be 130.2–262.5%, for famotidine, 102.5–117.8% for omeprazole, and 105.7–117.2% for omeprazole sulfone (Table 2).

**Table 2:** Validation results for the determination of famotidine, omeprazole, and omeprazole sulfone in equine plasma using UHPLC-MS/MS.

Analyte	LLOQ (ng/mL)	QC^1^ level	Nominal concentration (ng/mL)	Intra-day (*n* = 3)		Inter-day (*n* = 3)		Recovery ± RSD (%)	Matrix effect ± RSD (%)	Carryover
				Precision (%)	Accuracy (%)	Precision (%)	Accuracy (%)			
Famotidine	50	LLOQ	50	4.1	101.2	0.9	100.5	37.6 ± 4.5	262.5 ± 24.7	n.d.^5^
		LQC^2^	150	1.0	99.4	5.6	95.7	32.7 ± 6.0	162.5 ± 8.8	
		MQC^3^	400	9.3	100.9	2.6	101.0	35.5 ± 13.0	184.8 ± 1.9	
		HQC^4^	750	1.5	88.0	3.5	94.0	38.4 ± 18.1	130.2 ± 4.9	
Omeprazole	50	LLOQ	50	3.8	94.0	0.9	100.4	25.6 ± 6.1	108.7 ± 2.4	n.d.
		LQC	150	0.7	95.6	1.9	101.0	25.2 ± 4.6	109.8 ± 1.7	
		MQC	400	4.5	98.4	2.8	104.7	31.4 ± 9.1	117.8 ± 6.2	
		HQC	750	3.8	92.0	11.9	106.3	34.7 ± 10.9	102.5 ± 2.7	
Omeprazole sulfone	50	LLOQ	50	1.9	96.2	1.4	99.6	24.4 ± 4.1	105.7 ± 3.4	n.d.
		LQC	150	2.3	97.6	3.0	101.9	25.4 ± 5.0	108.7 ± 1.3	
		MQC	400	0.6	106.5	2.1	104.7	31.6 ± 5.1	117.2 ± 3.1	
		HQC	750	4.3	104.1	8.3	107.0	27.5 ± 6.6	112.4 ± 2.2	

QC^1^, Quality control; LQC^2^, Lower quality control; MQC^3^, Middle quality control; HQC^4^, High quality control; n.d.^5^, No detected.

## 4 Discussion

In this study, HPLC and UHPLC–MS/MS analytical methods were developed for the simultaneous determination of famotidine and omeprazole in equine plasma. During the method development process, sample preparation protocols were optimized to ensure efficient and reliable quantification of the target analytes within plasma matrix. The validity of these optimized methods was demonstrated through comprehensive method validation. Furthermore, the practical applicability of the established methods was successfully verified by applying them to the analysis of PK samples obtained from horses following drug administration.

The peak splitting of famotidine observed during HPLC analysis is attributed to the pKa of the analyte. Famotidine has a pKa of approximately 6.7, thus, under neutral analytical conditions, it exists in an equilibrium state where ionized and non-ionized forms coexist. Consequently, the observed split peaks are interpreted as the result of the chromatographic separation of these two distinct chemical species within the column. In reversed-phase chromatography, the highly polar ionized form exhibits weaker interactions with the stationary phase and thus elutes faster than the non-ionized form (42, 43). To validate this hypothesis, the ionization ratio of famotidine as a function of pH was theoretically calculated using the Henderson-Hasselbalch equation, and these values were compared with the experimental results obtained from adjusting the pH of the sample preparation solvent within the range of 2–6 (Table S1). Consistent with theoretical predictions, the ionized form predominated under strongly acidic conditions (pH 2–3), eluting as a single peak with a retention time faster than that of the non-ionized form. Conversely, as the pH increased toward basicity, a tendency for the non-ionized form to elute in increasing proportions was observed. Based on these findings, it was confirmed that the peak splitting of famotidine resulted from the mixture of ionized and non-ionized forms due to its pKa. Therefore, lowering the pH to a strongly acidic level provided both optimal peak shape and quantitative reliability. However, it is important to note that HPLC analysis relies on UV absorption characteristics, meaning detection depends on the absorbance response rather than the absolute quantity of the substance. Consequently, differences in extinction coefficients between the ionized and non-ionized forms can influence detection (44). Therefore, the ratio of the two peak areas observed in the chromatogram may exhibit a quantitative deviation from the theoretically calculated values, leading to potential discrepancies. Furthermore, no peak splitting of famotidine was observed in the UHPLC–MS/MS analysis, even without specific pH adjustment during sample preparation. This is attributed to the presence of FA in the mobile phase, which created an acidic environment that stabilized the analyte in a single ionized form. These findings significantly contributed to the establishment of the UHPLC–MS/MS method.

During the HPLC analysis, a deviation from linearity in the calibration curve of omeprazole was observed at concentrations exceeding 1 mg/mL, characterized by a distinct plateauing effect. This phenomenon is attributed to the solubility differences of omeprazole depending on the solvent composition. While omeprazole exhibits a high solubility of over 19 mg/mL in DMSO, which was used for preparing stock and working solutions, its solubility is significantly lower in water (approximately 0.5 mg/mL). Since plasma has a high-water content, it is hypothesized that omeprazole failed to remain fully dissolved at high concentrations, leading to partial precipitation and a consequent decrease in the slope of the calibration curve. To address this issue, a comparative experiment was conducted between the conventional method of adjusting the pH of the extraction solvent and a modified method involving direct pH adjustment of the matrix. PBS was utilized as a surrogate matrix for plasma in these experiments. The results indicated that the conventional approach failed to resolve the precipitation issue. However, direct adjustment of the matrix pH successfully restored linearity even at high concentrations. This outcome appears to be a consequence of the buffering capacity inherent in biological matrices such as plasma (45). It is postulated that indirect pH adjustment via the extraction solvent was insufficient to alter the matrix environment enough to increase the solubility of omeprazole. In contrast, directly modifying the pH of the matrix itself likely created a more favorable environment for the ionization of omeprazole, thereby effectively enhancing its solubility. Furthermore, although the optimized high-concentration range in this study may exceed typical therapeutic blood levels observed in PK studies, establishing linearity in this range is valuable. It provides insight into the physicochemical properties of the drug and the matrix and suggests that the developed method may be applicable in various fields, such as pharmaceutical formulation studies, where analysis of high concentration samples is often required (46–50).

Although the HPLC method was successfully developed and validated, it exhibited limitations in quantifying trace levels of famotidine and omeprazole in equine plasma. Although attempts were made to overcome these limitations by optimizing peak shape for famotidine and enhancing solubility for omeprazole, the method did not meet the sensitivity requirements necessary for PK studies. These optimization efforts provided critical insights into the physicochemical properties of the analytes and their behavior in the biological matrix. Notably, these findings indicate that analytical conditions optimized for HPLC are not directly transferable to UHPLC–MS/MS. Building upon this foundational research, a high-sensitivity UHPLC–MS/MS system was introduced. This transition resulted in a substantial improvement in analytical sensitivity. The LLOQ for both famotidine and omeprazole was 100 μg/mL using the HPLC method, whereas the UHPLC–MS/MS method achieved an LLOQ of 50 ng/mL for both analytes. Thus, the UHPLC–MS/MS method provided an approximately 2000-fold increase in analytical sensitivity compared with HPLC, enabling the successful quantitative analysis of trace levels of these drugs in equine plasma. The HPLC method was developed for the quantification of relatively high concentrations of famotidine and omeprazole, whereas the UHPLC–MS/MS method was developed for trace-level quantification and pharmacokinetic analysis. Accordingly, different validation scopes were applied based on the intended analytical purpose of each method. More comprehensive validation was performed for the UHPLC–MS/MS method considering its pharmacokinetic application, whereas the validation scope for the HPLC method was defined according to its intended application for high-concentration analysis.

In the HPLC analysis, a substantial carryover of 38.5% was observed for omeprazole, indicating a relatively high residual effect. The observed high carryover may affect quantitative accuracy and precision by increasing the analyte signal in subsequent samples. However, because the accuracy and precision results obtained during the validation in the present study have remained reproducible within acceptable ranges, the observed carryover has been considered unlikely to have a significant impact on the quantitative performance of the method under the evaluated conditions. In such cases, increasing the frequency or duration of autosampler needle washing or performing consecutive blank injections following the analysis of high-concentration samples may be considered as potential approaches for minimizing carryover. However, considering that no carryover was observed for the same analyte during UHPLC–MS/MS analysis, it is likely that this issue in the HPLC system can be sufficiently resolved by optimizing the injector conditions and selecting a more appropriate wash solvent. Regarding autosampler stability, the values obtained for omeprazole were somewhat elevated. However, as short-term stability remained within acceptable limits, these findings are interpreted as not being attributable to the intrinsic chemical instability of the analyte.

In the UHPLC–MS/MS method validation, the matrix effect for famotidine ranged from 130.2–262.5%, indicating significant ion enhancement. This phenomenon is attributed to the high polarity of famotidine, which results in a short retention time and subsequent co-elution with endogenous interfering substances, such as phospholipids, present in equine plasma. These matrix effects, including ion enhancement, can affect the ionization efficiency of the analyte and consequently alter the signal response, potentially leading to errors in the accuracy and precision of quantitative analysis. In this study, matrix-matched calibration was employed to minimize potential errors in quantitative accuracy and precision arising from matrix. In addition, the accuracy and precision results obtained during method validation have supported the quantitative reliability of the method. The revised Discussion has been updated to describe the potential impact of matrix effects on the accuracy and precision of quantitative analysis and the use of matrix-matched calibration as a strategy to mitigate this effect. While alternative extraction techniques such as solid-phase extraction (SPE) or liquid–liquid extraction (LLE) could effectively remove interfering substances and reduce matrix effects, they are often time-consuming and cost-prohibitive for PK studies requiring the rapid processing of large sample volumes. Therefore, PPT was selected as the optimal preparation method in this study due to its simplicity and high throughput efficiency. However, the relatively high matrix effect should be considered when assessing the robustness and transferability of the method. The extent of matrix effects may vary depending on the composition and source of the plasma matrix. Therefore, the matrix effect observed in equine plasma cannot be assumed to be consistent across different plasma sources. In addition, instrument configuration, chromatographic conditions, and ionization source conditions may differ across analytical laboratories, and these differences may further affect the matrix effect and the resulting analytical response. Therefore, these factors should be considered when transferring the method to different analytical laboratories. Extraction recovery was evaluated as relatively low, with values of 32.7–38.4% for famotidine, 25.6–34.7% for omeprazole, and 24.4–31.6% for omeprazole sulfone. This reduction is attributed to the absence of pH adjustment in the UHPLC–MS/MS extraction protocol, in contrast to the HPLC method. In HPLC analysis, pH adjustment could be applied because the analysis was conducted at relatively high concentrations. However, in UHPLC–MS/MS analysis of trace-level low concentrations, even the same degree of degradation may substantially reduce the detectable analyte signal (51, 52). This is also consistent with the chromatographic response observed under the tested extraction conditions (Figure 2). Therefore, pH adjustment was not applied to ensure the stability of the analytes, and these sample preparation conditions have contributed to the relatively low extraction recovery observed in the UHPLC-MS/MS analysis. Nevertheless, the method enabled trace-level quantification of all analytes by achieving an LLOQ of 50 ng/mL.

## 5 Application of the analytical method

The established analytical method was applied demonstrate its applicability to the pharmacokinetic analysis of famotidine and omeprazole in horses. The study was designed to compare the administration of famotidine alone (0.4 mg/kg, IV) and its co-administration with omeprazole (4 mg/kg, PO), and plasma concentration-time profiles were obtained to describe the pharmacokinetic characteristics under each administration condition. Plasma concentration-time curves were constructed from samples collected at predefined time points, and PK parameters were calculated using these profiles.

The PK parameters derived from the analysis are summarized as follows. For famotidine, the elimination half-life (*t_1/2_*) was 2.7 ± 0.7 h in the single-administration group and 2.3 ± 0.1 h in the co-administration group. The area under the plasma concentration-time curve from time zero to the last measurable concentration (*AUC_last_*) was calculated to be 1511.3 ± 363.4 ng·h/mL for single-administration and 1150.2 ± 22.6 ng·h/mL for co-administration. Clearance (*CL*) was 0.3 ± 0.1 L/h/kg and 0.3 ± 0.0 L/h/kg for single-administration and co-administration groups, respectively. The volume of distribution (*V_d_*) was determined to be 0.9 ± 0.03 L/kg for single-administration and 1.1 ± 0.05 L/kg for co-administration. For omeprazole, the PK profile following co-administration with famotidine was characterized and described in relation to previously reported literature values (53). Following co-administration, the maximum plasma concentration (*C_max_*) of omeprazole was 128.4 ± 76.0 ng/mL. The time to reach maximum plasma concentration (*T_max_*) was 1.0 ± 0.5 h, the t_1/2_ was 1.3 ± 0.4 h, and the *AUC_last_* to last measurable point was 251.4 ± 172.8 ng·h/mL. The apparent clearance (*CL/F*) and apparent volume of distribution (*V_d_/F*) were calculated as 23.2 ± 14.1 L/h/kg and 33.8 ± 11.96 L/kg, respectively. To evaluate the metabolic profile of omeprazole, its major metabolite, omeprazole sulfone, was also analyzed simultaneously. The *C_max_* was 54.8 ± 2.5 ng/mL, *T_max_* was 1.3 ± 0.3 h, the *t_1/2_* was 2.8 ± 0.8 h, and the *AUC_last_* was 188.8 ± 0.5 ng·h/mL. The *V_d_/F* was determined to be 70.84 ± 15.93 L/kg (Table 3).

**Table 3:** Pharmacokinetic parameters of famotidine, omeprazole, and omeprazole sulfone in horses following single administration of famotidine and co-administration with omeprazole (Mean ± SD).

Analyte	Group	*t*_1/2_^1^ (h)	*T*_max_^2^ (h)	*C*_max_^3^ (ng/mL)	*AUC*_last_^4^ (ng·h/mL)	*CL*^5^ (L/h/kg)	*CL*/*F*^6^ (L/h/kg)	*V*_d_^7^ (L/kg)	*V*_d_ /*F*^8^ (L/kg)
Famotidine	Single	2.7 ± 0.7	—	—	1511.3 ± 363.4	0.3 ± 0.1	—	0.9 ± 0.03	—
	Co-administration	2.3 ± 0.1			1150.2 ± 22.6	0.3 ± 0.0		1.1 ± 0.05	
Omeprazole	Single	13.0 ± 4.6^a^	2.2 ± 1.6^a^	1341.1 ± 604.5^a^	2884.7 ± 1007.9^a^	—	^a^1.6 ± 0.7	—	^a^30.1 ± 16.7
	Co-administration	1.3 ± 0.4	1.0 ± 0.5	128.4 ± 76.0	251.4 ± 172.8		23.2 ± 14.1		33.8 ± 11.96
Omeprazole sulfone	Co-administration	2.8 ± 0.8	1.3 ± 0.3	54. ±2.5	188.8 ± 0.5	—	—	—	70.84 ± 15.93

t_1/2_^1^, half-life; T_max_^2^, Time to reach maximum plasma concentration; C_max_^3^, Maximum plasma concentration; AUC_last_^4^, Area under the curve to the last measurable concentration; CL^5^, Clearance; CL/F^6^, Apparent clearance; V_d_^7^, Volume of distribution; V_d_ /F^8^, Apparent volume of distribution; ^a^Re-evaluation of the regulation of omeprazole in racehorses: An evidence-based approach (49).

**Figure 4:**
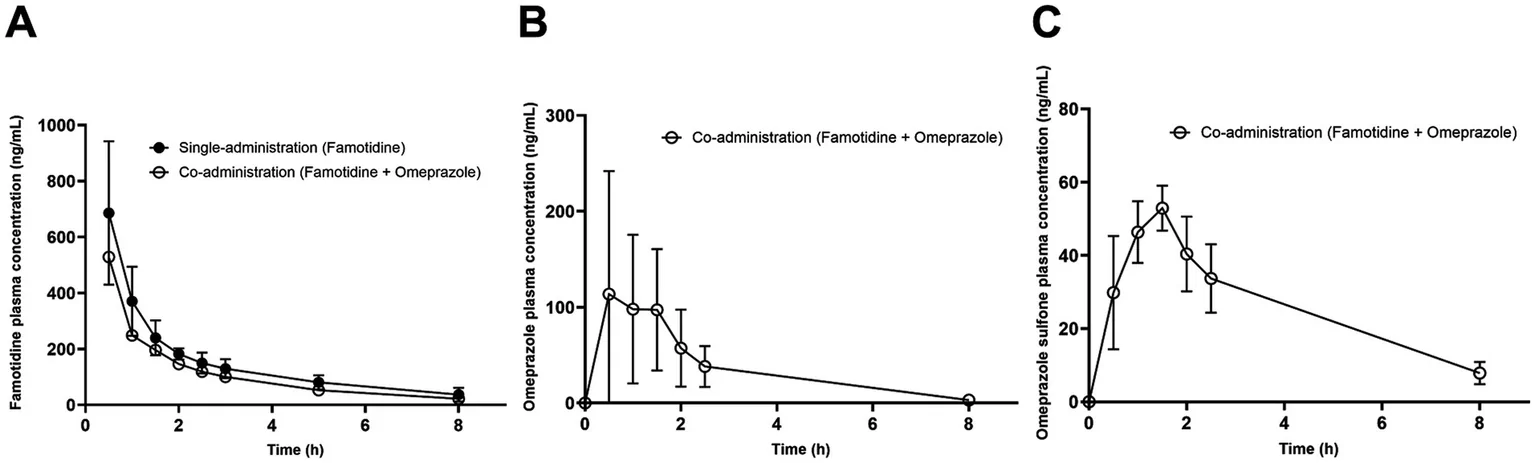
Mean plasma concentration-time profiles of famotidine, omeprazole, and omeprazole sulfone in horses following single or co-administration. **(A)** Famotidine following single intravenous administration (0.4 mg/kg) and co-administration with oral omeprazole (4 mg/kg), **(B)** Omeprazole following co-administration, and **(C)** Omeprazole sulfone following co-administration. Data are expressed as mean standard deviation (SD) (*n* = 2 for each administration group).

Upon applying the developed UHPLC–MS/MS simultaneous analysis method to plasma samples obtained from drug-administered horses, quantitative analysis was performed to construct plasma concentration-time curves and calculate PK parameters. The plasma concentration-time profile of famotidine exhibited a typical elimination pattern following IV administration (Figure 4A). In contrast, high inter-individual variability was observed in the PK profiles of omeprazole (Figure 4B). Following co-administration, omeprazole concentrations rapidly reached peak levels but displayed large standard deviations at each time point, indicating substantial variability in the observed plasma exposure. Previous studies have suggested that inter-individual differences in physiological factors, including gastrointestinal pH and gastric emptying, may contribute to variability in the oral disposition of omeprazole by affecting its exposure to acidic gastric conditions and, consequently, its bioavailability (54). Furthermore, omeprazole undergoes extensive metabolism by CYP enzymes, and inter-individual differences in metabolic activity may also contribute to variability in systemic exposure (55, 56). These mechanistic explanations are consistent with previously reported pharmacological characteristics of omeprazole. However, they should be regarded as plausible hypotheses rather than definitive explanations of the variability observed in the present study. The present dataset directly demonstrates substantial inter-individual variability in omeprazole plasma concentrations, whereas the relative contributions of physiological factors, absorption, and metabolism require confirmation in future pharmacokinetic studies with a larger sample size.

The simultaneous detection of omeprazole sulfone demonstrated that the metabolite was formed following oral administration with a peak concentration observed at approximately 1.3 h post-dose (Figure 4C). This finding is consistent with active metabolic conversion of omeprazole during the observed pharmacokinetic period. However, this data does not establish whether inter-individual differences in omeprazole sulfone formation contributed to the observed variability in omeprazole plasma concentrations. Further pharmacokinetics studies with a larger sample size and more comprehensive sampling would be required to clarify these mechanisms. These findings should be interpreted cautiously given the small sample size and inherent inter-individual variability.

Several limitations should be considered when interpreting the pharmacokinetic findings of the present study. The animal experiment was designed primarily as a PoC evaluation of the applicability of the developed analytical method to equine plasma samples rather than as a statistically powered comparative pharmacokinetic study. The small number of animals (*n* = 4), with two animals in each administration group, limits the statistical power of the PK evaluation and the generalizability of the observed pharmacokinetic profiles. Therefore, the PK findings should be considered preliminary, and further studies involving a larger number of animals are warranted to confirm these observations and enable more robust pharmacokinetic comparisons.

## 6 Conclusion

In this study, a dual-platform analytical strategy based on HPLC and UHPLC–MS/MS was developed and validated for the simultaneous determination of famotidine, omeprazole, and the major metabolite, omeprazole sulfone, in equine plasma. Analytical challenges, including peak splitting of famotidine and the limited solubility of omeprazole, were successfully addressed through tailored pH control during sample preparation. While the HPLC method effectively monitored high-concentration ranges, the UHPLC–MS/MS method provided a 2000-fold sensitivity improvement (LLOQ: 50 ng/mL), providing the sensitivity required for trace-level pharmacokinetic analysis. The validated methods were successfully applied to plasma samples from horses, demonstrating their applicability to pharmacokinetic observations. Importantly, this study demonstrates that analytical conditions optimized for HPLC are not directly transferable to UHPLC–MS/MS due to differences in analyte stability and sensitivity, highlighting the necessity of platform-specific sample preparation strategies. Overall, the developed methods provide a practical and sensitive analytical framework for the reliable determination of famotidine, omeprazole, and omeprazole sulfone in equine plasma.

## Data Availability

The original contributions presented in the study are included in the article/Supplementary material, further inquiries can be directed to the corresponding author.
