# Supplementary material for: A complementary dual-platform analytical strategy for resolving matrix effects and stability limitations in bioanalysis
Source: Front Vet Sci. 2026 Sep 7;13:1951616. doi: 10.3389/fvets.2026.1951616 (PMC13590423; doi:10.3389/fvets.2026.1951616)
Supplement: Supplementary file 1 [file Table_1.DOCX]

Supplementary Material

# 1 Supplementary Tables

## 1.1 Supplementary Tables

**Supplementary Table 1.** Calculated percentages of the ionized and non-ionized forms of famotidine at various pH values.

| **pH value** | **% Ionized** | **% Non-ionized** |
| --- | --- | --- |
| pH 1 | 100.00 | 0.00 |
| pH 2 | 100.00 | 0.00 |
| pH 3 | 99.98 | 0.02 |
| pH 4 | 99.80 | 0.20 |
| pH 5 | 98.04 | 1.96 |
| pH 6 | 83.37 | 16.63 |
| pH 7 | 33.39 | 66.61 |
| pH 8 | 4.77 | 95.23 |
| pH 9 | 0.50 | 99.50 |

## The theoretical percentages were calculated using the Henderson-Hasselbalch equation based on the reference pKa value of famotidine (pKa ≈ 6.7)
